# Supplementary material for: Structural basis for the inhibition of translation through eIF2α phosphorylation
Source: Nat Commun. 2019 Jun 14;10:2640. doi: 10.1038/s41467-019-10606-1 (PMC6572841; doi:10.1038/s41467-019-10606-1)
Supplement: Supplementary file 1 — Supplementary Information [file 41467_2019_10606_MOESM1_ESM.pdf]

Supplementary Information

Supplementary Figure 1. 3D- classification scheme.

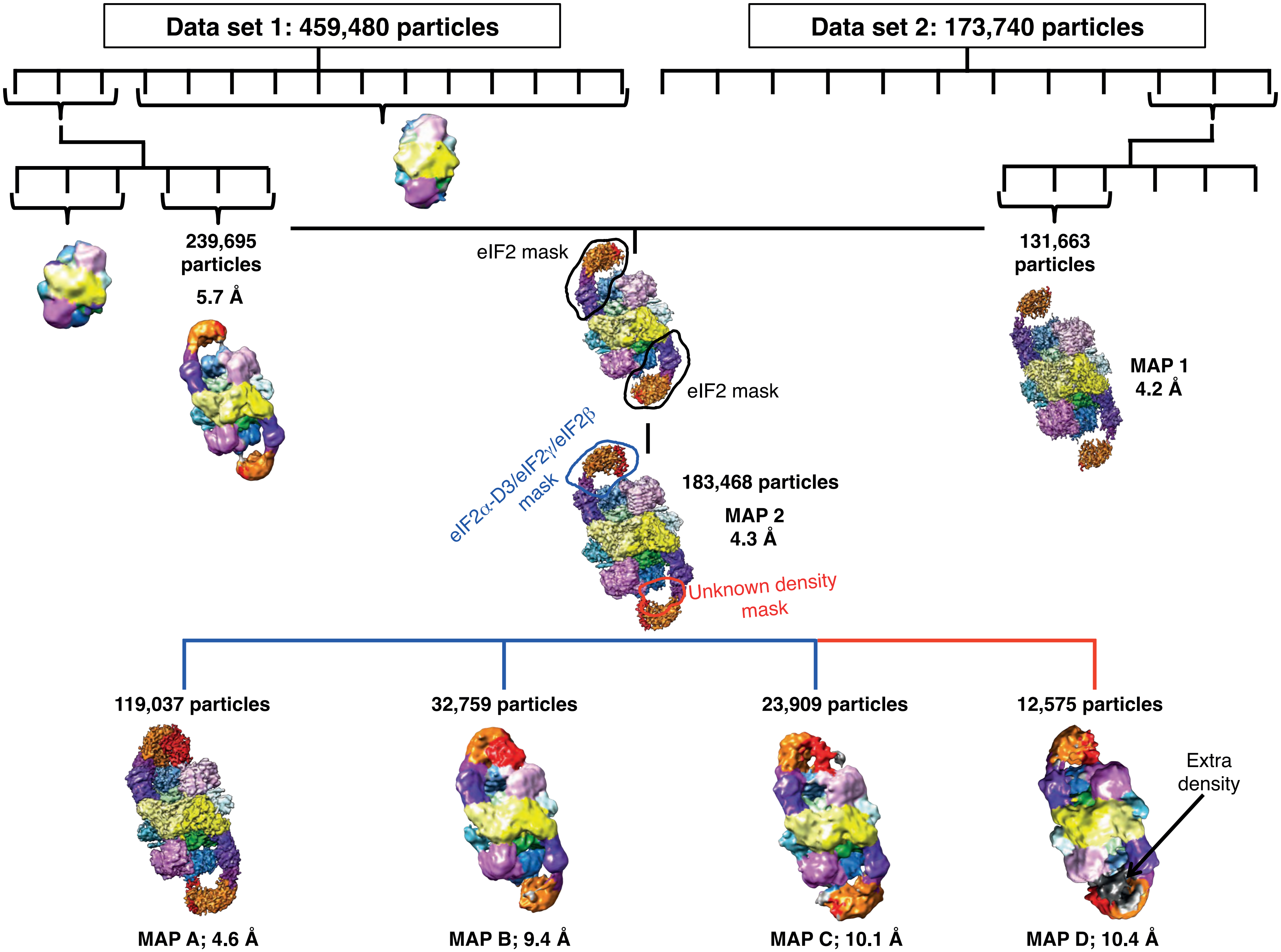

Supplementary Figure 2. Local resolutions of eIF2B - eIF2(αP) complex.

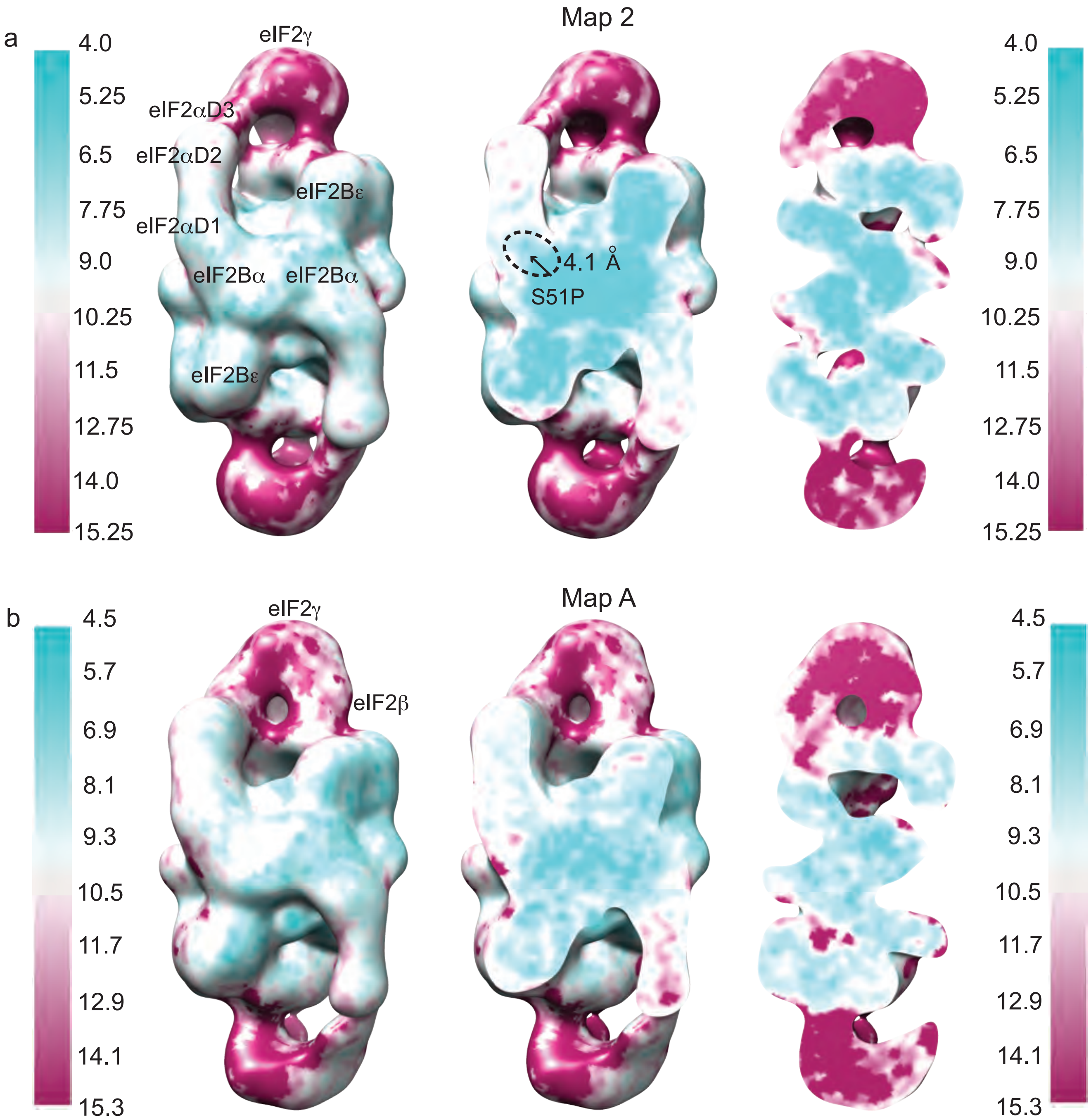

(a) Local resolutions of eIF2B - eIF2(αP) complex in map 2 (related to Fig. 1a) showing surface (left) and two cross-sections around eIF2α-D1 binding site and location of Ser-51(52 sequence numbering).

(b) Same as in (a), but in in map A (related to Fig. 1b).

Supplementary Figure 3. Superposition of the ISRIB bound human eIF2B to eIF2B - eIF2(αP) complex.

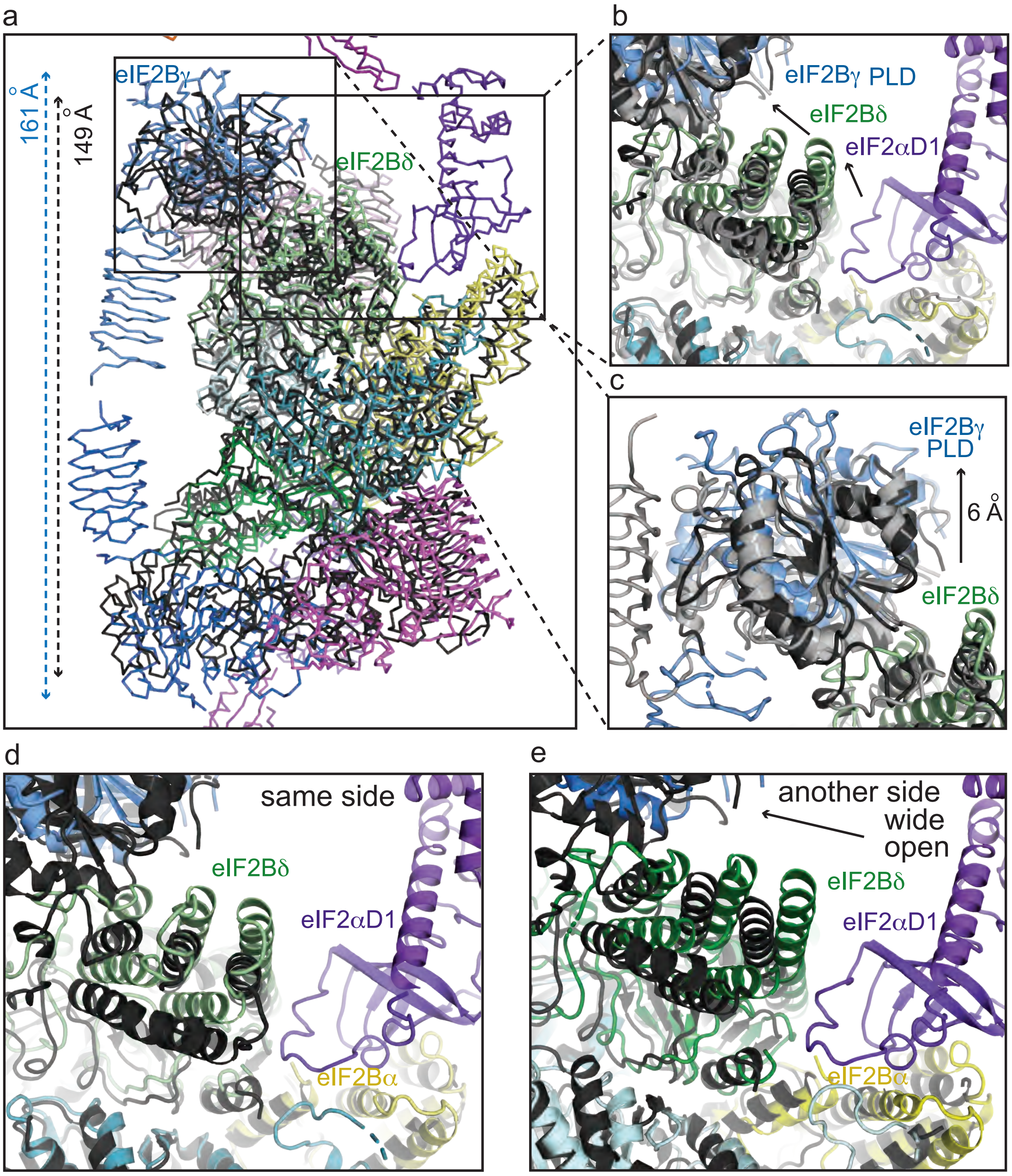

(a) Superposition of eIF2B-eIF2(αP) complex with human ISRIB bound eIF2B (black) (PDB 6CAJ) showing elongation of eIF2B hetero-decamer towards catalytic poles upon binding of eIF2 by ~ 12 Å.

(b) Same superposition as in (a), but also including S. pombe eIF2B structure (grey) (PDB 5B04), shows that elongation of eIF2B is mainly induced by closure eIF2B α and δ around eIF2α-D1 displacing eIF2Bγ outwards.

(c) Same superposition as in (a), showing displacement of eIF2Bγ in eIF2B-eIF2(αP) complex in one of the eIF2B poles.

(d) The binding site of eIF2α-D1 contacting the superposed eIF2Bα subunit showing reduced contacts with eIF2Bδ and local rearrangement of eIF2Bα interacting helices (indicated with an arrow).

(e) The other binding site of eIF2α-D1 showing that on this side the binding pocket for eIF2α-D1 formed by eIF2B α and δ is wide open.

Supplementary Figure 4. Structural model fitting of eIF2α-D1 in a density map.

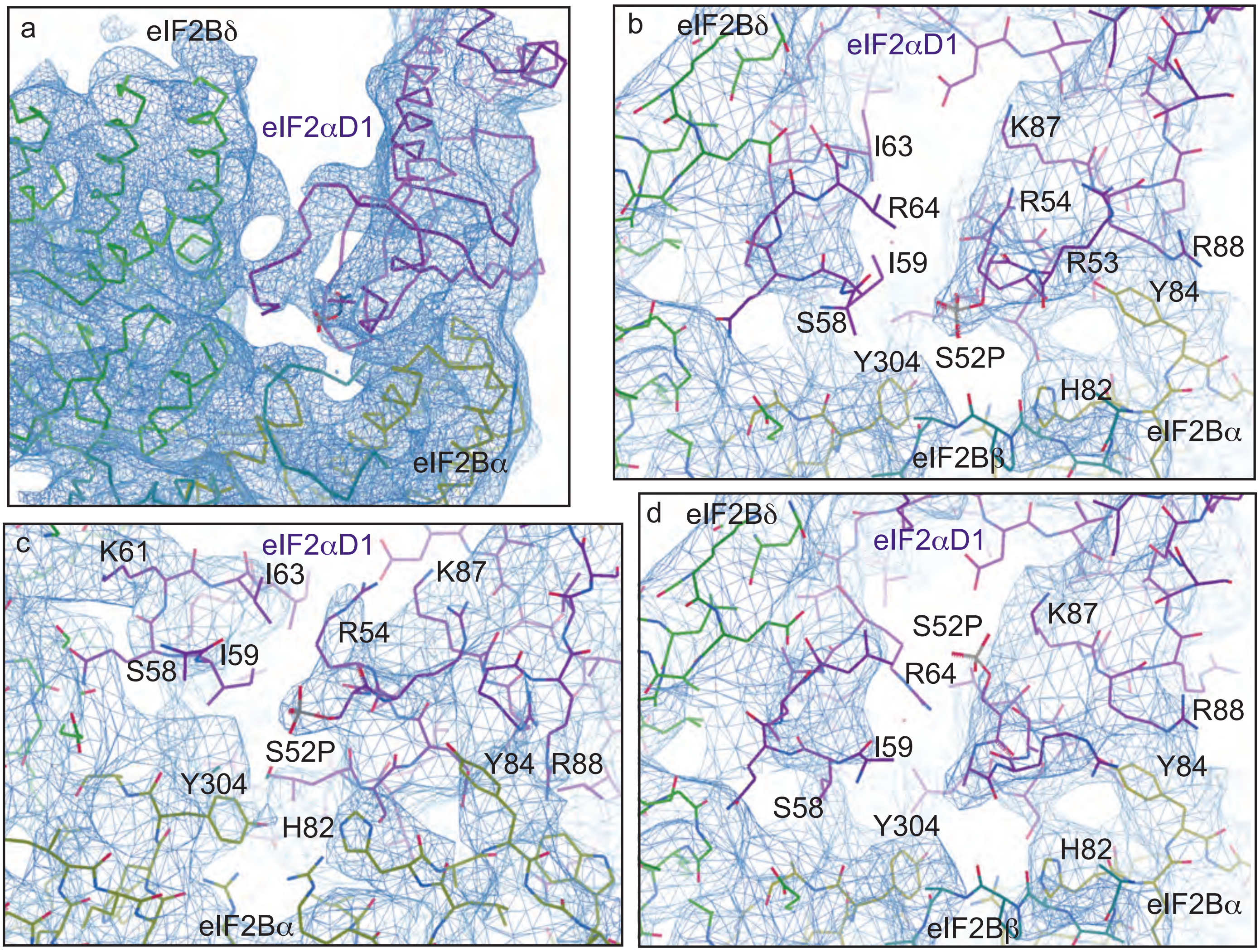

(a) Overall density for the contact of eIF2α-D1 with eIF2B α and δ subunits.

(b) Close view of the density around Ser51(P) (S52 sequence numbering) and our structural model fitting into the density.

(c) Same as in (b) but at different orientation.

(d) Fitting of a structural model as in reference 1 of the same region as in (b) in our density map.

Supplementary Figure 5. Examples of structural models fitting in density maps.

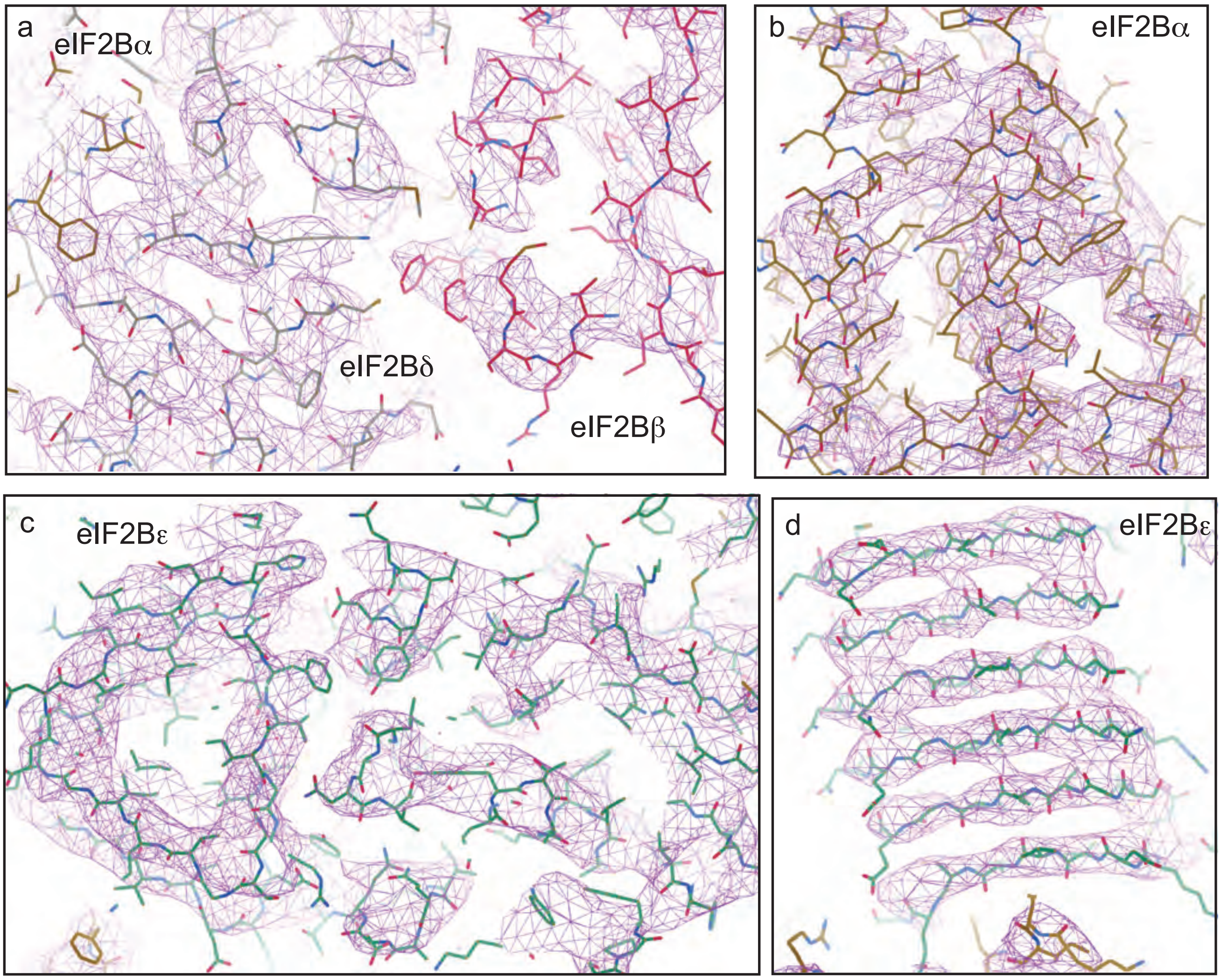

(a) Density for eIF2Bδ in contact with eIF2Bβ and eIF2Bα subunits.

(b) Density for eIF2Bα subunit.

(c) Density for eIF2Bε PLD domain.

(d) Density for eIF2Bε LβH domain.

Supplementary Figure 6. Multiple sequence alignment of eIF2B regulatory subunits using Clustal Omega.

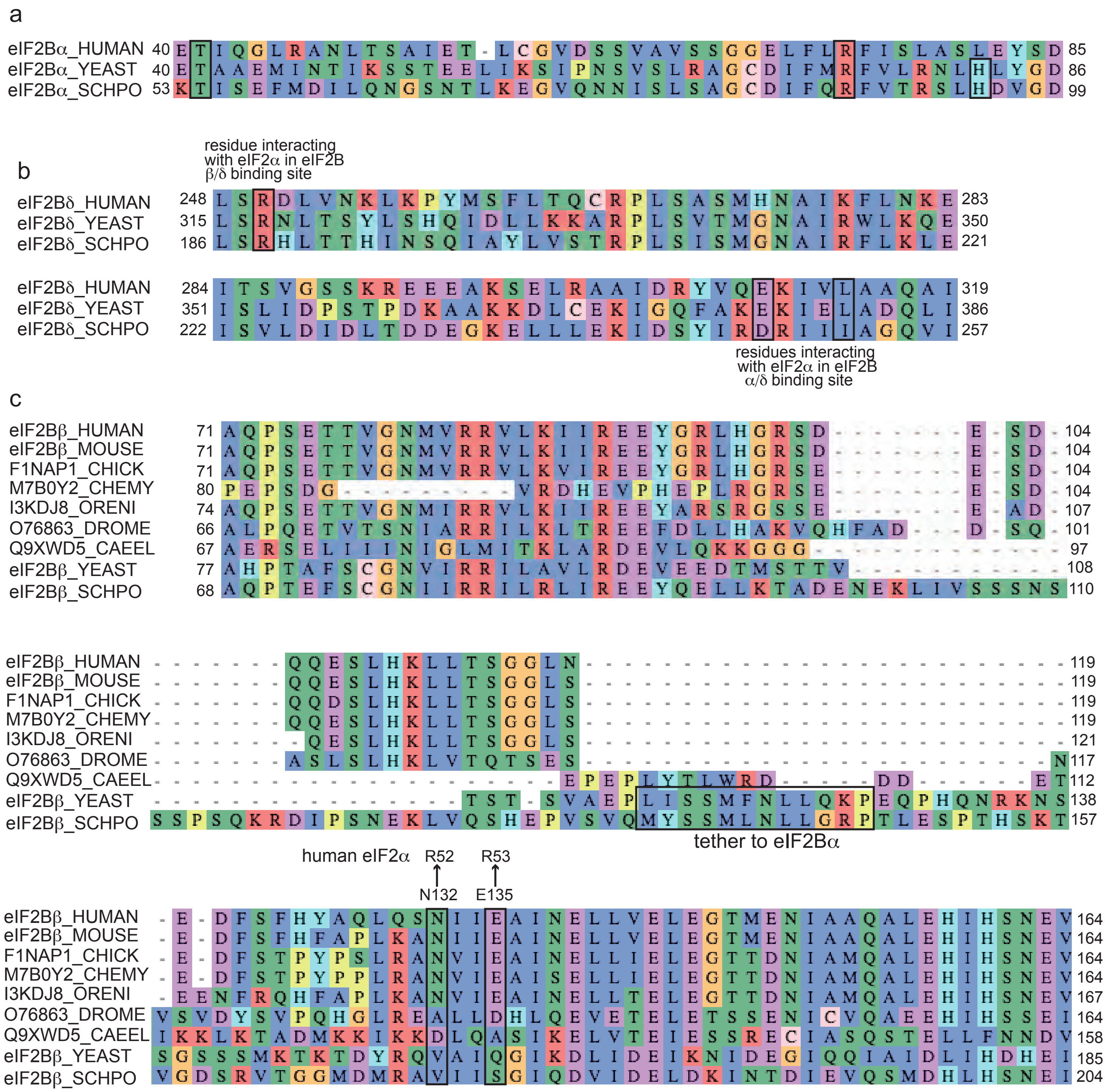

(a) Alignment of human, S. cerevisiae and S. pombe eIF2Bα sequences in the region of eIF2α binding site between α and δ subunits of eIF2B. Residues important for the interaction with eIF2α in ref.2 and this paper are boxed.

(b) Alignment of human, S. cerevisiae and S. pombe eIF2Bδ sequences in the region of both eIF2α binding sites. Residues that important for the interaction with eIF2α in references 2 and 3, and this paper are boxed.

(c) Alignment of human (Homo sapiens), mouse (Mus musculus), chicken (Gallus gallus), green sea-turtle (Chelonia mydas), fish (Oreochromis niloticus), fruit fly (Drosophila melanogaster), Caenorhabditis elegans, S. cerevisiae and S. pombe eIF2Bβ sequences in the region of alternative eIF2α binding site between β and δ subunits of eIF2B. Residues important for the interaction in in this site based on the structures of human eIF2B-eIF2 complex (refs 2, 3) are boxed. Alignment shows conservation of these residues (N132 and E135 according to human numbering) in vertebrates, however they are not conserved in yeast. Also part of the loop - tether (boxed), which in yeast interacts with eIF2Bα, is missing in the species where alternative binding site evolved. Drosophila melanogaster lost the tether, however alternative binding site is not entirely conserved.

Supplementary Figure 7. Biochemical and MS analysis of eIF2B-eIF2α(P) complex.

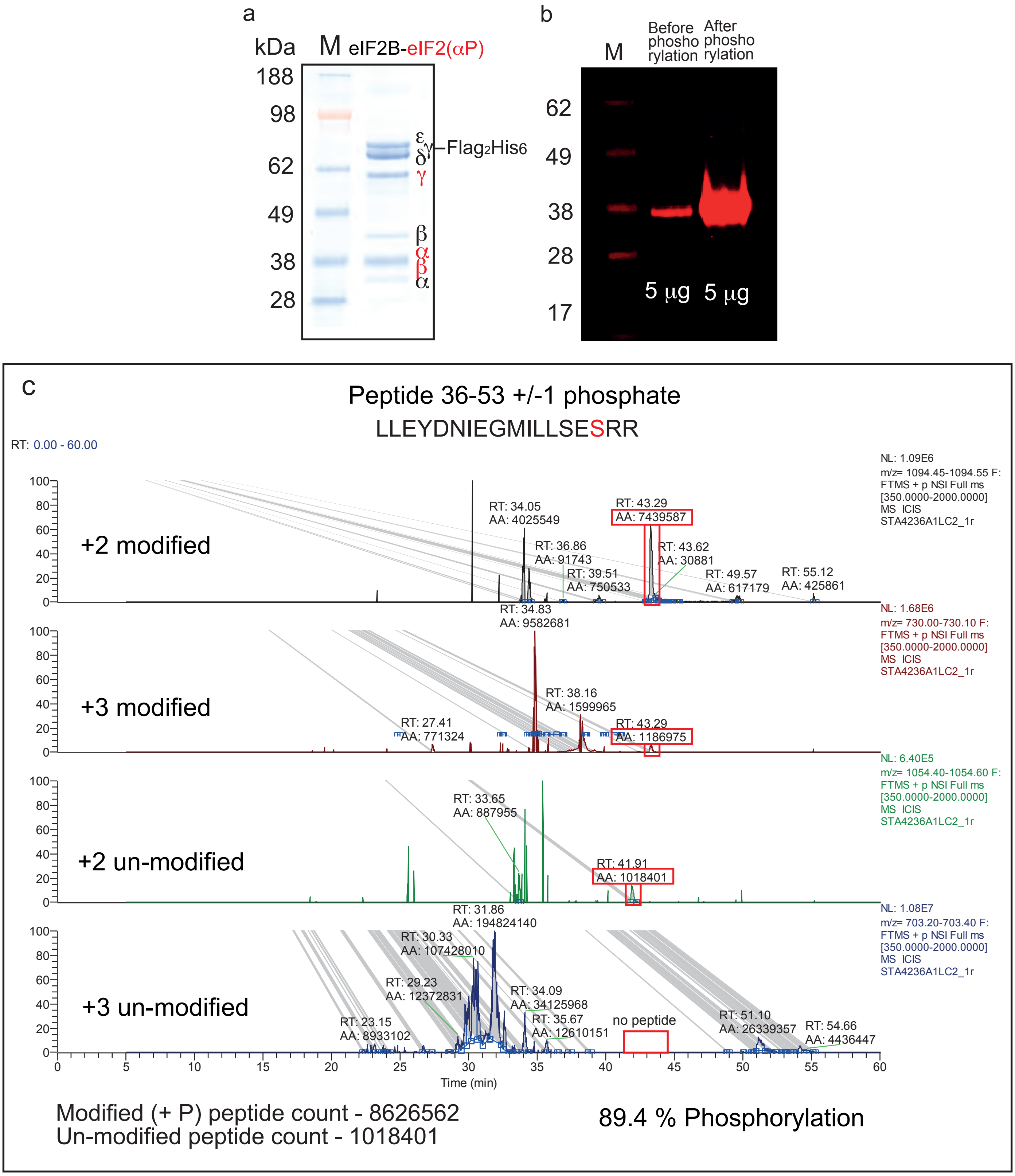

(a) SDS PAGE of eIF2B - eIF2(αP) complex with eIF2B subunits labelled in black and eIF2 subunits in red.

(b) Western blotting of eIF2 using antibodies specific against human eIF2α(P) (Invitrogen 44-728G) before and after phosphorylation with PKR (5 μg of eIF2 protein was loaded in each lane).

(c) Mass spectra of the 2+ and 3+ charge states of eIF2α peptide 36-53 (LLEYDNIEGMILLSESRR) after eIF2 was phosphorylated with PKR. Phosphorylation (89.4 %) was calculated based on the area of the peaks of modified (phosphorylation) and non-modified peptides in both charge states.

Supplementary Figure 8. Cryo-EM methods.

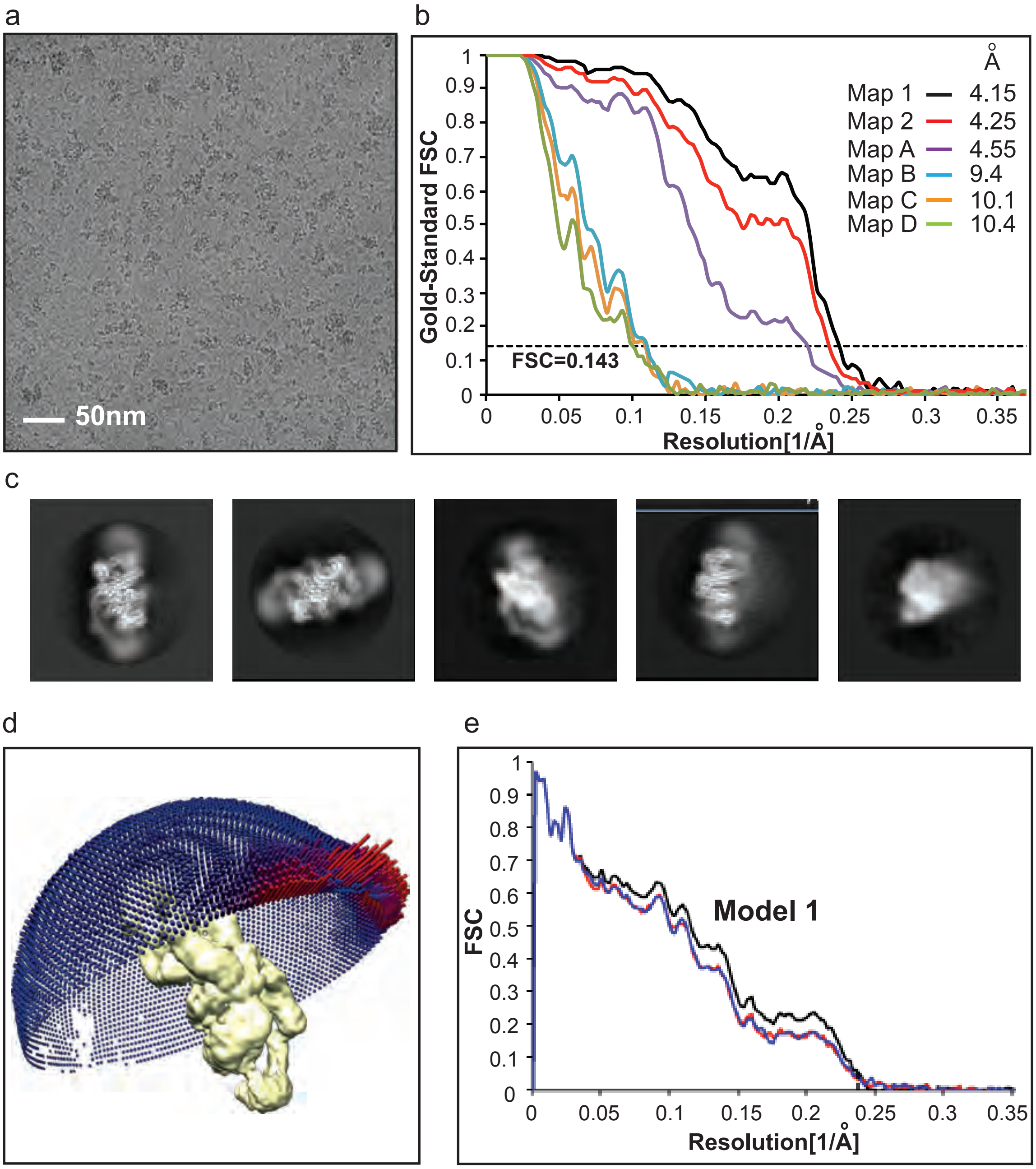

(a) Typical micrograph of eIF2B - eIF2(αP) complex (see methods for details).

(b) FSC curves.

(c) Representative 2-D classes after 2-D classification of eIF2B - eIF2(αP) complex.

(d) Orientation distribution plot for the eIF2B-eIF2 complex map from dataset I. Efficiency of the reconstruction measured with cryoEF[4] is 0.62 (good > 0.8; bad < 0.5) which shows that the complex suffers from some preferred orientation.

(e) FSC curve of the model versus map.

1. Adomavicius, T., Guaita M., Zhou Y, Jennings, M.D., Latif Z, Roseman, A.M., Pavitt, G.D. The structural basis of translational control by eIF2 phosphorylation. *BioRxiv, doi: https://doi.org/10.1101/501411* (2018).
2. Kenner, L. R., Anand, A.A., Nguyen, H.C., Myasnikov, A.G., Klose, C.J., McGeever, L.A., Tsai, J.C., MillerVedam, L.E., Walter, P., Frost, A. eIF2B-catalyzed nucleotide exchange and phosphoregulation by the integrated stress response. *Science* **364**, 491-495 (2019).
3. Kashiwagi, K., Yokoyama, T., Nishimoto, M., Takahashi, M., Sakamoto, A., Yonemochi, M., Shirouzu, M., Ito, T. Structural Basis for eIF2B Inhibition in Integrated Stress Response. *Science* **364**, 495-499 (2019).
4. Naydenova, K. & Russo, C. J. Measuring the effects of particle orientation to improve the efficiency of electron cryomicroscopy. *Nat Commun.* **8**, 629 (2017).
