# Supplementary material for: Structural basis for the inhibition of translation through eIF2α phosphorylation
Source: Nat Commun. 2019 Jun 14;10:2640. doi: 10.1038/s41467-019-10606-1 (PMC6572841; doi:10.1038/s41467-019-10606-1)
Supplement: Supplementary file 3 — Reporting Summary [file 41467_2019_10606_MOESM3_ESM.pdf]

nature research

Corresponding author(s): NCOMMS-18-38205-A

Last updated by author(s): May 3, 2019

Reporting Summary

Nature Research wishes to improve the reproducibility of the work that we publish. This form provides structure for consistency and transparency in reporting. For further information on Nature Research policies, see Authors & Referees and the Editorial Policy Checklist.

Statistics

For all statistical analyses, confirm that the following items are present in the figure legend, table legend, main text, or Methods section.

n/a	Confirmed	
☒	☐	The exact sample size (n) for each experimental group/condition, given as a discrete number and unit of measurement
☒	☐	A statement on whether measurements were taken from distinct samples or whether the same sample was measured repeatedly
☒	☐	The statistical test(s) used AND whether they are one- or two-sided<br>*Only common tests should be described solely by name; describe more complex techniques in the Methods section.*
☒	☐	A description of all covariates tested
☒	☐	A description of any assumptions or corrections, such as tests of normality and adjustment for multiple comparisons
☒	☐	A full description of the statistical parameters including central tendency (e.g. means) or other basic estimates (e.g. regression coefficient) AND variation (e.g. standard deviation) or associated estimates of uncertainty (e.g. confidence intervals)
☒	☐	For null hypothesis testing, the test statistic (e.g. F, t, r) with confidence intervals, effect sizes, degrees of freedom and P value noted<br>*Give P values as exact values whenever suitable.*
☒	☐	For Bayesian analysis, information on the choice of priors and Markov chain Monte Carlo settings
☒	☐	For hierarchical and complex designs, identification of the appropriate level for tests and full reporting of outcomes
☒	☐	Estimates of effect sizes (e.g. Cohen's d, Pearson's r), indicating how they were calculated

Our web collection on statistics for biologists contains articles on many of the points above.

Software and code

Policy information about availability of computer code

Data collection	Automated cryo-EM data collection was carried out using EPU software (FEI-Thermofisher)
Data analysis	MotionCor 2, Gctf, Relion 2, ResMap, Chimera, Coot 0.8, Refmac 5.8, ProSMART, Molprobity, Pymol, CryoEF.

For manuscripts utilizing custom algorithms or software that are central to the research but not yet described in published literature, software must be made available to editors/reviewers. We strongly encourage code deposition in a community repository (e.g. GitHub). See the Nature Research guidelines for submitting code & software for further information.

Data

Policy information about availability of data

All manuscripts must include a data availability statement. This statement should provide the following information, where applicable:

- Accession codes, unique identifiers, or web links for publicly available datasets
- A list of figures that have associated raw data
- A description of any restrictions on data availability

The data that support the findings of this study are available from the corresponding author upon reasonable request. PDB and EMDB codes are available in https://www.rcsb.org/ and https://www.ebi.ac.uk/pdbe/emdb/, respectively. The codes are EMDB: 4543, EMDB: 4544, EMDB: 4545, EMDB: 4546, EMDB: 4547, EMDB: 4548, for Map 1, Map 2, Map A, Map B, Map C and Map D, respectively. Six atomic coordinate models have been deposited in the PDB with accession codes PDB: 6QG0, PDB: 6QG1, PDB: 6QG2, PDB: 6QG3, PDB: 6QG5 and PDB: 6QG6 for Maps 1, 2, Map A, Map B, Map C and Map D, respectively

Field-specific reporting

Please select the one below that is the best fit for your research. If you are not sure, read the appropriate sections before making your selection.

☒ Life sciences ☐ Behavioural & social sciences ☐ Ecological, evolutionary & environmental sciences

For a reference copy of the document with all sections, see nature.com/documents/nr-reporting-summary-flat.pdf

Life sciences study design

All studies must disclose on these points even when the disclosure is negative.

Sample size	If by sample size we mean number of particles in a CryoEM data collection, then total number of particles were selected using Relion software. References for template-based particle picking were obtained from 2D class averages that were calculated from particles semi-automatically picked with EMAN2 from a subset of the micrographs.
Data exclusions	Particles excluded belonged to classes not well 2D-aligned.
Replication	Two datasets from two different sample preparations were recorded, giving similar results.
Randomization	Not relevant in a cryo-EM study
Blinding	Not relevant in a cryo-EM study

Reporting for specific materials, systems and methods

We require information from authors about some types of materials, experimental systems and methods used in many studies. Here, indicate whether each material, system or method listed is relevant to your study. If you are not sure if a list item applies to your research, read the appropriate section before selecting a response.

Materials & experimental systems

n/a	Involved in the study	
☒	☐	Antibodies
☒	☐	Eukaryotic cell lines
☒	☐	Palaeontology
☐	☒	Animals and other organisms
☒	☐	Human research participants
☒	☐	Clinical data

Methods

n/a	Involved in the study	
☒	☐	ChIP-seq
☒	☐	Flow cytometry
☒	☐	MRI-based neuroimaging

Animals and other organisms

Policy information about studies involving animals; ARRIVE guidelines recommended for reporting animal research

Laboratory animals	Saccharomyces cerevisiae, strains GP3511 and GP4109
Wild animals	*Provide details on animals observed in or captured in the field; report species, sex and age where possible. Describe how animals were caught and transported and what happened to captive animals after the study (if killed, explain why and describe method; if released, say where and when) OR state that the study did not involve wild animals.*
Field-collected samples	*For laboratory work with field-collected samples, describe all relevant parameters such as housing, maintenance, temperature, photoperiod and end-of-experiment protocol OR state that the study did not involve samples collected from the field.*
Ethics oversight	*Identify the organization(s) that approved or provided guidance on the study protocol, OR state that no ethical approval or guidance was required and explain why not.*

Note that full information on the approval of the study protocol must also be provided in the manuscript.
